# Biostimulant Capacity of an Enzymatic Extract From Rice Bran Against Ozone-Induced Damage in *Capsicum annum*

**DOI:** 10.3389/fpls.2021.749422

**Published:** 2021-11-19

**Authors:** Sandra Macias-Benitez, Salvadora Navarro-Torre, Pablo Caballero, Luis Martín, Elisa Revilla, Angélica Castaño, Juan Parrado

**Affiliations:** Departamento de Bioquímica y Biología Molecular, Facultad de Farmacia, Universidad de Sevilla, Seville, Spain

**Keywords:** ozone, ROS, rice bran, enzymatic extract, MAPK

## Abstract

Ozone is a destructive pollutant, damaging crops, and decreasing crop yield. Therefore, there is great interest in finding strategies to alleviate ozone-induced crop losses. In plants, ozone enters leaves through the stomata and is immediately degraded into reactive oxygen species (ROS), producing ROS stress in plants. ROS stress can be controlled by ROS-scavenging systems that include enzymatic or non-enzymatic mechanisms. Our research group has developed a product from rice bran, a by-product of rice milling which has bioactive molecules that act as an antioxidant compound. This product is a water-soluble rice bran enzymatic extract (RBEE) which preserves all the properties and improves the solubility of proteins and the antioxidant components of rice bran. In previous works, the beneficial properties of RBEE have been demonstrated in animals. However, to date, RBEE has not been used as a protective agent against oxidative damage in agricultural fields. The main goal of this study was to investigate the ability of RBEE to be used as a biostimulant by preventing oxidative damage in plants, after ozone exposure. To perform this investigation, pepper plants (*Capsicum annuum*) exposed to ozone were treated with RBEE. RBEE protected the ozone-induced damage, as revealed by net photosynthetic rate and the content of photosynthetic pigments. RBEE also decreased the induction of antioxidant enzyme activities in leaves (catalase, superoxide dismutase, and ascorbate peroxidase) due to ozone exposure. ROS generation is a common consequence of diverse cellular traumas that also activate the mitogen-activated protein kinase (MAPK) cascade. Thus, it is known that the ozone damages are triggered by the MAPK cascade. To examine the involvement of the MAPK cascade in the ozone damage *CaMPK6-1*, *CaMPK6-2*, and *CaMKK5* genes were analyzed by qRT-PCR. The results showed the involvement of the MAPK pathway in both, not only in ozone damage but especially in its protection by RBEE. Taken together, these results support that RBEE protects plants against ozone exposure and its use as a new biostimulant could be proposed.

## Graphical Abstract

Explanatory diagram of the protective action of RBEE against the oxidizing agent ozone.

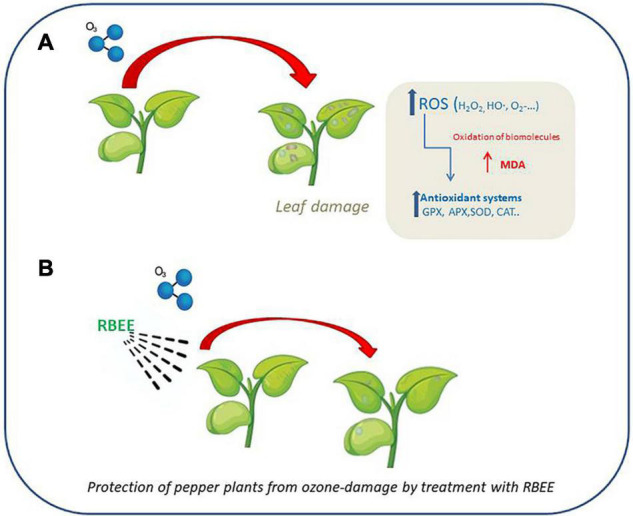

## Introduction

Ozone (O_3_) is a destructive pollutant with negative effects on human and ecosystem health that induces abiotic stress in plants – affecting photosynthetic carbon assimilation, stomatal conductance, and plant growth – that damages crops and decreases crop yield (Ainsworth et al., 2012).

In plants, O_3_ enters through the stomata and is degraded into secondary reactive oxygen species (ROS), including H_2_O_2_, O_2_^⋅–^, and HO^⋅^, in the apoplastic space (Vainonen and Kangasjärvi, 2015). High levels of ROS can lead to ROS stress that causes direct or indirect ROS-mediated damage on a variety of molecules including lipid peroxidation in cellular membranes, protein denaturation, carbohydrate oxidation, and pigment breakdown (Sharma et al., 2012). Even more, ROS stress can eventually lead to changes in gene expression and even cell death. However, ROS are generated in the metabolism of plant cells and therefore plants need systems that scavenge oxidizing species. Constitutive enzymatic and non-enzymatic systems in cells protect them from ROS-induced damage and are inducible under biotic and abiotic stress that triggers a rapid increase in ROS, including O_3_ exposure (Sachdev et al., 2021). Enzymatic ROS-scavenging mechanisms include antioxidant enzymes such as superoxide dismutase (SOD), catalase (CAT), ascorbate peroxidase (APX), glutathione peroxidase (GP), guaiacol peroxidase (GPX) and dehydroascorbate reductase (DHAR) (Gill and Tuteja, 2010).

Specific ROS also act as “second messengers” in signal transduction pathways that lie downstream initial event in cells. The mitogen-activated protein kinase (MAPK) cascade is one of the signaling pathways sensitive to the cell’s redox status. Therefore ROS generation is a common consequence of diverse cellular traumas that activate the MAPK cascade and studies in different plants have demonstrated that O_3_ exposure activates some components of the MAPK cascade (Marcus et al., 2000; Liu et al., 2015; Liu and He, 2017).

Studies on the effect of O_3_ on agricultural productivity showed an estimated 5–15% crop yield loss in the United States (Avnery et al., 2011) and a 5% in Europe (Mills et al., 2007). To alleviate the drop in crop productivity, the main defense strategy over the past four decades has been the application of chemicals, mainly synthetic antioxidants (fungicides, insecticides, herbicides, nematocides, growth regulators, antitranspirants, antioxidants from the rubber industry, etc.) (Didyk and Blum, 2010). These xenobiotic products have varied protection capacity with some producing ineffective or even detrimental side effects. The most effective product was found to be EDU (ethylene diurea– (*N*-[2-(2-oxo-1-imidazolidinyl) ethyl]-N0 phenylurea), developed by the duPont Chemical company (Archambault et al., 2000) whose main mechanism of action is not yet clear. Currently, more effective alternatives are being sought, such as extracts of plant origin, which do not generate environmental toxicity.

Rice Bran is a by-product of rice (*Oryza sativa*) milling that retains most of the bioactive compounds present in rice grain. These bioactives include naturally occurring antioxidants, mainly γ-oryzanol, tocopherols, tocotrienols, and polyphenols. Besides that, rice bran is also a good source of protein and fat (Goufo and Trindade, 2014). However, the low bioavailability of its biopolymers and bioactive compounds due to its high insolubility is the main drawback when considering rice bran as a biostimulant. Using enzymatic technology, our group has developed a process that enables obtaining a stable, water-soluble rice bran enzymatic extract (RBEE) from rice bran. RBEE has shown a high content in bioactive compounds, particularly phytosterols, γ–oryzanol, and tocols, and an increased content in peptides and free amino acids (Santa-María et al., 2010).

According to its content in bioactive compounds, RBEE has shown both *in vivo* and *in vitro* functional properties such as antioxidant capacity (Santa-María et al., 2010), hypocholesterolemic activity (Revilla et al., 2009), and antiproliferative and inmunoactivatory abilities (Revilla et al., 2013). Beneficial effects found in cells and animal models, especially those derived from its antioxidant capacity, lead us to consider the use of RBEE as a biostimulant in agriculture.

This work aims to evaluate the biostimulant capacity of RBEE. To this end, we propose studying the role of RBEE in plant protection against abiotic stress mediated by ROS, specifically in O_3_ exposure. We chose pepper plants (*Capsicum annuum*) since pepper is a vegetable crop of great agricultural and economic importance being the second most traded spice in the world. Heavy losses in pepper production are frequently caused by abiotic stresses including O_3_ exposition, in fact *Capsicum* pepper cultivation is almost entirely located in regions where O_3_ concentration is increasing to phytotoxic levels (Bortolin et al., 2016). For these reasons pepper plants are an interesting research target to evaluate the negative effects of O_3_.

Pepper plants exposed to O_3_ were treated with RBEE and antioxidant enzyme activities and specific functional parameters were analyzed. Treatment with RBEE improved physiological parameters, such as net photosynthetic rate and photosynthetic pigments content in plants exposed to with O_3_. RBEE treatment also decreased the induction of antioxidant enzyme activities in leaves (CAT, SOD, GPX, and APX) due to O_3_ exposure.

Additionally, to search whether MAPK cascade is involved in the protective role of RBEE against O_3_-induced damage we analyzed the transcriptional expression of *CaMPK6-1*, *CaMPK6-2*, and *CaMKK5* genes. The high expression of genes studied strongly support that RBEE protective capacity involved the MAPK signaling cascade.

## Materials and Methods

### Rice Bran Enzymatic Extract Preparation

Rice bran (*O. sativa* var. *indica*) raw material was provided by Herba Ricemills, S.L.U (Sevilla, Spain). Rice bran is obtained during the polishing/milling of raw rice grains once their husks have been stripped. Rice bran was processed by enzymatic hydrolysis using the hydrolytic agent (Biocom, Spain) subtilisin (EC 3.4.21.62), a protease from *Bacillus licheniformis* as hydrolytic agent (Biocom, Spain) in a bioreactor with controlled temperature (60°C) and pH (pH 8), using the pH-stat method (Parrado et al., 2006). The processing of this product follows different steps, including solid separation and concentration. The final product RBEE is a brown syrup that is completely water-soluble.

Rice bran enzymatic extract bran macro and micronutrient composition was characterized as previously described (Parrado et al., 2006).

### Plant Treatment

*Capsicum annum* L. var. *grossum* (pepper) plants were raised from seeds in plastic pots containing an organic commercial substrate (Gramoflor GmbH und Co., KG.) and Osmocote^®^ (NPK 15-9-12), and grown inside the University of Seville Glasshouse General Services on a phytoclimatic chamber, with a controlled temperature of 18–22°C, 50% relative humidity, adequate irrigation with tap water and a photoperiod of 16 h light (1200 μmol.m^–2^. s^–1^)/8 h darkness. After 8 days of transplantation, 20 pepper plants were selected and divided in 4 treatments (5 plants per treatment): control plants (C), plants treated with RBEE, control plants under O_3_ exposition (C + O_3_), and plants treated with RBEE under O_3_ exposition (RBEE + O_3_).

To evaluate the protection capacity of the treatment with RBEE, RBEE and RBEE + O_3_ plants were foliar sprayed with an aqueous solution of RBEE at 0.1% a total of four times at 5-day intervals. At the same time, control plants (C and C + O_3_) were sprayed with distilled water the same times. After 5 days of the last spray treatment, C + O_3_ and RBEE + O_3_ plants were transferred to another phytoclimatic chamber with an O_3_ generator (ZONOSISTEM GM 5000 O3 Generator) attached and exposed to three consecutive fumigations with 100 ppB of O_3_ for 6 h (from 10:00 a.m. to 4:00 p.m.). After O_3_ fumigation, plants of all treatments were sprayed again with the corresponding solution (RBEE 0.1% or distilled water).

Finally, 24 h after the last exposure to O_3_, foliar samples were taken from each plant and the analyses described below were carried out.

### Physiological Status in Plants

#### Determination of Net Photosynthetic Rate

Twenty-four hours after the las ozone treatment, the net photosynthetic rate (A_N_) was measured in plants using an IRGA (LI-6400XT, LI-COR Inc., Nev., EEUU) with a light chamber for the leaf (Li-6400-02B, Li-Cor Inc.). Measurements (*n* = 20) were performed between 10 a.m. and 2 p.m. hours under a photosynthetic photon flux density of 1500 μmol.m^–2^.s^–1^, a deficit of vapor pressure of 2–3 kPa, a temperature around 25°C, and a CO_2_ concentration environment of 400 μmol.mol^–1^ air. Each measurement was recorded after the stabilization of the exchange of gases was equilibrated (120 s).

#### Chlorophyll Content in Leaves

Chlorophyll content was extracted from random leaves from plants of each treatment. Fifty milligrams of leaves were homogenized in acetone 100% (v/v) and saline solution 0.9% (w/v) using a homogenizer (Hiscox and Israelstam, 1979). The total chlorophyll content was determined at 652 nm by using the absorbance coefficient of extinction 34.5 cm^–1^⋅ μg^–1^ (Arnon, 1949):


$${\text{Abs}}_{652nm}=\left[\text{chlorophyll}\right]\cdot 34.5{\mathrm{cm}}^{-1}\cdot \mu g^{-1}.$$

#### Delayed Fluorescence Measurements

Delayed fluorescence (DF) was detected using a plant imaging system (NightShade LB 985, Berthold Technologies, Germany) equipped with a deeply cooled CCD camera according to López-Jurado et al. (2020). From plants of each treatment, 2–3 intact leaves of approximately the same size were separated and placed in the plant imaging system. The leaves were illuminated for 20 s with light supplied from far red (730 nm), red (660 nm), green (565 nm), and blue (470 nm) LED panels at 2, 105, 40, and 110 μmol.m^–2^.s^–1^, respectively. Immediately after the LEDs were turned off, DF was measured, and the recorded intensities of light were converted to counts per second (cps). Data were then normalized to each leaf area to obtain comparable cps values across treatments.

### Oxidative Stress Evaluation in Plants

#### Determination of Lipid Peroxidation

To quantify lipid peroxidation in leaf homogenates malondialdehyde (MDA) content was determined using the thiobarbituric acid reactive substances (TBARS) assay (Esterbauer and Cheeseman, 1990). Samples of 1 g of leaves were homogenized with 2 ml of 0.1% trichloroacetic acid (TCA). The homogenates were centrifuged at 8000 × *g* for 10 min at 4°C, and the supernatants were filtered through a 0.2 mm aseptic filter. Then, 0.3 ml of each sample was mixed with 0.9 ml of 20% TCA containing 0.5% TBA. The solutions were heated at 95°C for 1 h, immediately cooled, and centrifuged as above. Finally, the absorbance at 532 and 600 nm of the supernatants was recorded, and MDA concentration was calculated by subtracting the non-specific absorption at 600 nm from the absorption at 532 nm by using the absorbance coefficient of extinction 156 mM^–1^ cm^–1^. The results obtained were expressed in nmol.g^–1^ fresh weight (FW).

#### Antioxidant Enzymes Analyses

To observe the stress of plants, enzymatic activities of APX, SOD, guaiacol peroxidase (GPX), and CAT were measured as described by Duarte et al. (2015). Briefly, vegetal extract was extracted in extraction buffer (50 mM sodium phosphate buffer; pH 7.6) from 500 mg of leaves. Catalase activity was determined at 240 nm in a reaction solution containing the assay buffer (50 mM sodium phosphate buffer, pH 7.0) and 100 mM H_2_O_2_. APX activity was assayed in the assay buffer with 12 mM H_2_O_2_ and 0.25 mM L-ascorbate and measured at 290 nm. SOD activity was determined by monitorization of the pyrogallol oxidation at 325 nm by the addition of 3 mM pyrogallol. Guaiacol peroxidase activity was measured at 470 nm in a reaction mixture containing the assay buffer, 2 mM H_2_O_2_, and 20 mM guaiacol. To determine the auto-oxidation of the substrates, control assays were performed in absence of enzymatic extract samples (Duarte et al., 2015). Finally, the total protein content in the enzymatic extracts was measured according to Bradford (1976).

#### Oxidative Stress Index

Oxidative stress index (OSI) is a parameter that expresses the global oxidative stress in plants (Pérez-Palacios et al., 2017). It was calculated with the formula described in Paredes-Páliz et al. (2018). Values greater than 1 indicated that the leaves were stressed, whereas values less than 1 indicated that the leaves were without oxidative stress.

### Plant RNA Extraction and qRT-PCR Assay

RNA was extracted from 100 mg of leaves using RNeasy^®^ Plant Mini Kit (Qiagen, Germany) following the manufacturer’s instructions. Then, RNA samples were treated with DNA-free^TM^ kit (ThermoFisher, United States) to remove the residual DNA. Immediately, RNA samples were retrotranscripted to cDNA using QuantiTect^®^ Reverse Transcription kit (Qiagen, Germany) according to the manufacturer’s instructions, and the expression of *CaMPK6-1*, *CaMPK6-2* (*Arabidopsis* orthologs AtMPK6, Liu et al., 2015), and *CaMKK5* (*Arabidopsis* orthologs AtMKK5, Liu et al., 2015) genes were analyzed by qPCR by triplicate. qPCR was performed using SensiFAST^TM^ SYBR^®^ No-ROX kit (Bioline, France) and primers described in Table 2 following the supplier’s instructions, in a LightCycler^®^ 480 II thermo-cycler (Roche, Switzerland) under the next conditions: 95°C for 2 min and 50 cycles at 95°C for 5 s, followed by 60°C for 10 s, and finally 72°C for 15 s. The beta-tubulin housekeeping gene was used to normalize results from different samples. Primers used in these assays are described in Table 1. Expression signals were quantified and normalized using LightCycler^®^ 480 Software version 1.5 (Roche). The expression fold was calculated according to Livak and Schmittgen (2001):


ΔCq=AVECq(TargetAssay)-AVECq(ReferenceAssay)

ΔΔCq=ΔCq(TestSample)-ΔCq(ReferenceSample)

$$\text{RQ}=2^{-\Delta \Delta \text{Cq}}.$$

**TABLE 1:** Primers for qRT-PCR amplifications.

**Protein**	**Accession numbers**	**Primer**	**Sequence**	**References**
Beta tubulin	EF495259.1	*BTF*	5′-GAGGGTGAGTGAGCAGTTC-3′	Guo et al., 2012
		*BTR*	5′-CTTCATCGTCATCTGCTGTC-3′	
CaMPK6-1	CA08g04480	*MK6-1F*	5′-AAAGCCTCTGTTTCCTGGTAG-3′	Liu et al., 2015
		*MK6-1R*	5′-CTCCTTCTGGGATCAAATGTC-3′	
CaMPK6-2	CA03g14570	*MK6-2F*	5′-CAGAGATCATGTACACCA-3′	
		*MK6-2R*	5′-TCGCACCTGTTATTCTCCTTCTG-3′	
CaMKK5	CA03g36820	*MKK5F*	5′-GATTTCATTGCCTGCTGTTTG-3′	
		*MKK5R*	5′-GTGCCTGATGGACCTGATTAC-3′	

**TABLE 2:** Analytical composition of RBEE.

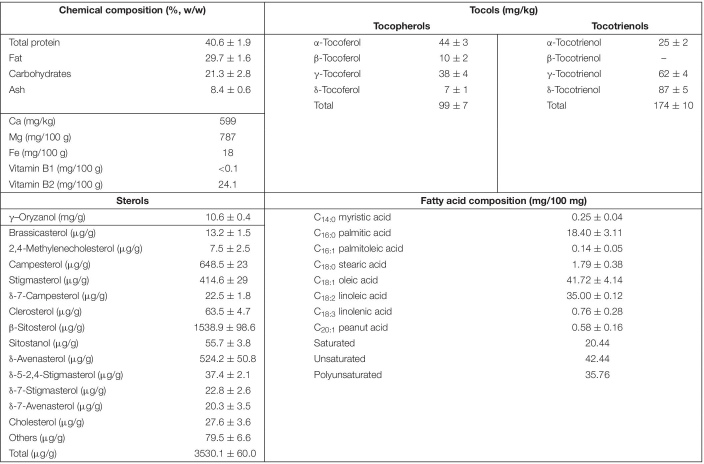

*All data are presented as mean ± SD of three independent experiments.*

### Statistical Analysis

Statistical analysis was performed using Statistica software version 6.0 (StatSoft Inc.). First, the normality was checked by the Kolmogorov–Smirnov test. The means of the different treatments were compared using two-way ANOVA and the statistic differences were carried out by Tukey test (*F*-test).

## Results

### Rice Bran Enzymatic Extract Chemical Characterization

The enzymatic process of obtaining the new water-soluble biostimulant (RBEE) from rice bran has been previously shown (Parrado et al., 2006). The biological tool involved in the enzymatic process for obtaining the RBEE is subtilisine (EC 3.4.21.62). This protease extracts, solubilizes, and hydrolyzes the initial insoluble proteins in brans, reducing the size of original rice bran proteins to soluble peptides. This process also led to the solubilization of hydrophobic compounds as lipids and bioactive metabolites in an emulsion (w/o). The chemical composition is shown in Table 2.

The two major components in RBEE are the proteins and lipids in equal amounts. Protein fraction is mainly comprised of peptides <5 kDa (see Supplementary Figure SM1). These peptides interact with the lipid fraction that allows fatty acids and hydrophobic bioactive compounds such as polyphenols, phytosterols, tocopherols, and tocotrienols (Revilla et al., 2009) to be soluble in water, leading to a greater bioavailability.

Rice bran is rich in prominent bioactives such as phytosterols. Interestingly, our RBEE retains major phytosterols described in rice bran (Santa María et al., 2016), the most abundant phytochemical found being γ–oryzanol (Table 2).

Other bioactive molecules such as tocopherols and tocotrienols (vitamin E) are also present in rice bran. Seven homologs of tocols were identified in RBEE (α-, β-, γ-, and δ-tocopherols and α-, γ-, and δ-tocotrienols). Among these, γ-tocotrienols were the most abundant, followed by α-tocopherols and α-tocotrienols (Table 2).

Rice bran enzymatic extract components also include polyunsaturated fatty acids, so linoleic acid and linolenic acid constitute 35.76% of total fatty acid content (Table 2). Finally, RBEE also contains flavonoids (flavonols, 0.62 mg/g; flavanols, 2.90 mg/g) and phenolic acids (20.14 mg/g).

Accordingly, the enzymatic process increases the bioavailability of RB without losing bioactive components.

### Physiological Status in Plants

After the experiments, the physiological status in pepper plants was determined by assaying diverse parameters, such as net photosynthetic rate, chlorophyll content, and DF.

**FIGURE 1:**
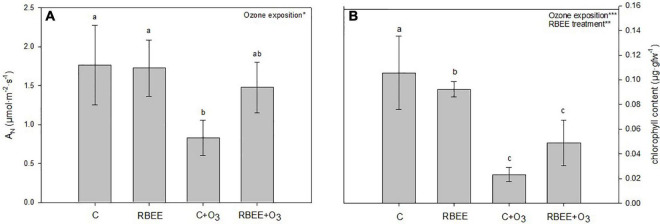
Physiological parameters. **(A)** Net photosynthetic rate (A_N_) and **(B)** total chlorophyll content in pepper plants in response to two conditions of ozone (O_3_) (0 and 100 ppm) under a treatment without and with rice bran enzymatic extract (RBEE). Values represent mean ± SD, *n* = 5. Different letters indicate means that are significantly different from each other (two-way ANOVA, O_3_ exposition × RBEE treatment; HSD test, *P* < 0.05). O_3_ exposition and RBEE treatment in the corner of the panel indicate main or interaction significant effects (*P < 0.05; **P < 0.01; ***P < 0.0001).

The net photosynthetic rate was significantly affected by O_3_ exposure (Figure 1A). After the treatments with RBEE, plants showed similar values to the control plants indicating that the RBEE does not interfere with the photosynthesis. However, when the plants were exposed to O_3_ this rate significantly decreased. This decrease was recovered by 88% in the plants exposed to O_3_ and treated with RBEE.

The total chlorophyll content in plants was significantly affected by both O_3_ exposure and RBEE treatment (Figure 1B). Leaves of control plants treated with ozone showed 4.6-folds less content of total chlorophyll than non O_3_-exposed control plants. However, despite RBEE treatment did not recover the content of total chlorophyll under O_3_ conditions, this treatment improved this content by 53%.

**FIGURE 2:**
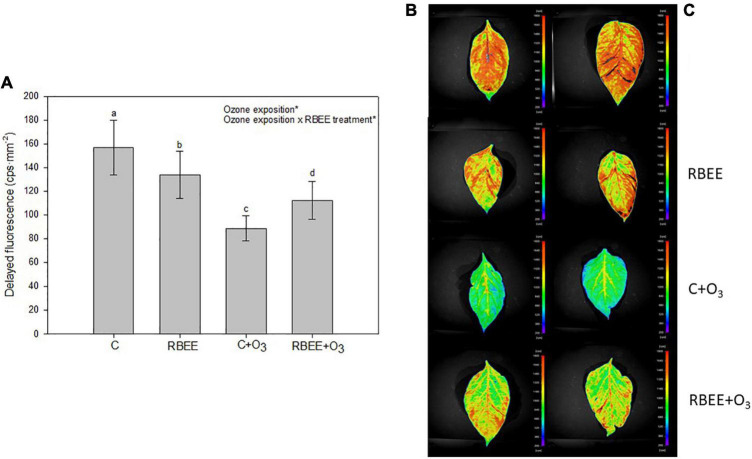
Delayed fluorescence in leaves of pepper plants in response to two conditions of ozone (O_3_) (0 and 100 ppm) under a treatment without and with rice bran enzymatic extract (RBEE). **(A)** Counts per second (cps) values for each treatment. Values represent mean ± SD, *n* = 5. Different letters indicate means that are significantly different from each other (two-way ANOVA, O_3_ exposition × RBEE treatment; HSD test, *P* < 0.05). O_3_ exposition and ozone exposition × RBEE treatment in the corner of the panel indicate main or interaction significant effects (*P < 0.0001); **(B)** photographs taken by the plant imaging system NightShade LB 985. The color scale mirrors the detected counts per second (cps) of delayed fluorescence emission in leaves.

To monitor plant stress status, DF closely related to photosynthetic reactions and chlorophyll content was also measured (Zhang et al., 2019). Both RBEE treatment and O_3_ exposure produced a significant decrease in DF emissions. The lowest measurements were detected in untreated, O_3_-fumigated plants, and the RBEE and RBEE + O_3_ plants showed a less substantial decrease in DF emissions than the C + O_3_ plants (Figures 2A,B). Although O_3_ also produced a loss of DF signals in the RBEE-treated plants, this decrease was much less marked compared to C + O_3_ plants, highlighting a lower stress state of these plants.

These results suggest that RBEE protects plants against photosynthetic damages by O_3_ exposure and maintain the physiological status of plants under these conditions. With the naked eye, although with less significant differences, we could also observe the attenuation of the visible foliar symptoms caused by ozone in the plants treated with RBEE before ozonization. C + O_3_ plants showed a more widespread chlorosis, as well as the appearance of small brown spots that were not present on RBEE + O_3_ plants (Supplementary Figure SM2).

### Oxidative Stress Level in Plants

**FIGURE 3:**
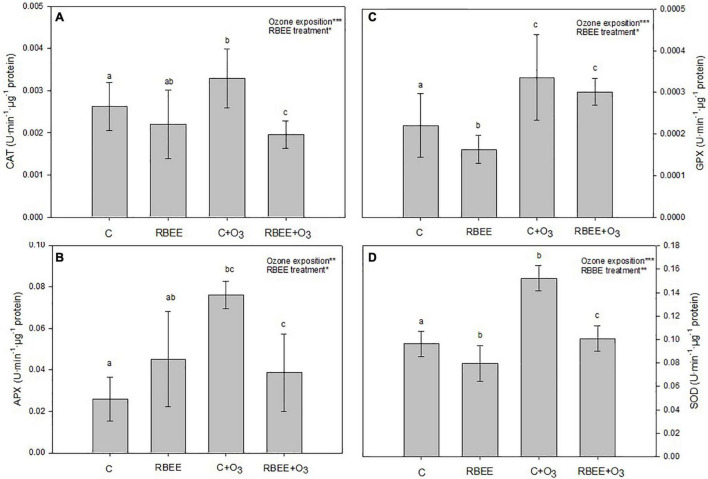
Antioxidant enzyme activities. **(A)** Catalase (CAT), **(B)** ascorbate peroxidase (APX), **(C)** guaiacol peroxidase (GPX), and **(D)** superoxide dismutase (SOD) activities from leaves of pepper plants in response to two conditions of ozone (O_3_) (0 and 100 ppm) under a treatment without and with rice bran enzymatic extract (RBEE). Values represent mean ± SD, *n* = 5. Different letters indicate means that are significantly different from each other (two-way ANOVA, O_3_ exposition × RBEE treatment; HSD test, *P* < 0.05). O_3_ exposition and RBEE treatment in the corner of the panels indicate main or interaction significant effects (*P < 0.01; **P < 0.001; ***P > 0.0001).

To observe the oxidative stress after O_3_ exposure and RBEE treatment, antioxidant enzyme activities were measured. Both variables had significant effects on these activities (Figure 3). As expected, O_3_-exposed plants showed a significant increase in their enzymatic activities in comparison with the control to alleviate the oxidative stress caused by ozone, underline that SOD and APX showed more activity than the other enzymes. Nevertheless, the activity of enzymes decreased both in an O_3_ environment and when plants were treated with RBEE but no exposed to O_3_, with the insignificant exception of the APX activity without ozone exposure (Figure 3B). In absence of O_3_, the activities decrease 16, 26, and 17%, in CAT, GPX, and SOD, respectively. In O_3_-exposed plants, the decreases were 41, 49, 10, and 34% in CAT, APX, GPX, and SOD, respectively, being the APX activity the most affected.

**FIGURE 4:**
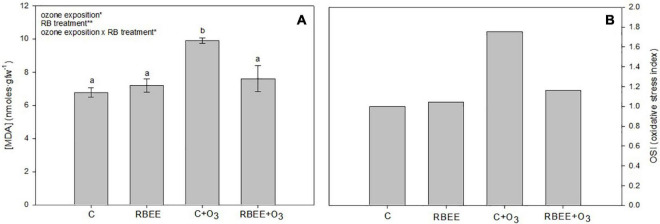
Malondialdehyde concentration (MDA) **(A)** and oxidative stress index (OSI) **(B)** in leaves of pepper plants in response to two conditions of ozone (O_3_) (0 and 100 ppm) under a treatment without and with rice bran enzymatic extract (RBEE). Values represent mean ± SD, *n* = 5. Different letters indicate means that are significantly different from each other (two-way ANOVA, O_3_ exposition × RBEE treatment; HSD test, *P* < 0.05). O_3_ exposition, RBEE treatment, and O_3_ exposition × RBEE treatment in the corner of the panels indicate main or interaction significant effects (*P < 0.01; **P < 0.001).

To check the lipid peroxidation of plants, the content of MDA, commonly used as a marker of oxidative stress (Oszlányi et al., 2020), was measured in our leaf samples. As shown in Figure 4A, higher concentrations of MDA were recorded in the C + O_3_ plants, which presented a significant increase in MDA content compared to all other experimental plants. On the other hand, the plants sprayed with RBEE showed similar levels of lipid peroxidation to those of the control plants, showing the protective effect of RBEE treatment against oxidative stress caused by O_3_ exposure.

All these positive effects of RBEE in plants in an O_3_ environment were also reflected in the OSI (Figure 4B). The OSI shows that O_3_-exposed plants treated with RBEE contain more similar stress levels to that of the control plants. These results support the protective effects of RBEE against O_3_-induced oxidative damage. Furthermore, RBEE application in leaves did not produce any oxidative stress in pepper plants.

### Mitogen-Activated Protein Kinase Genes Expression

**FIGURE 5:**
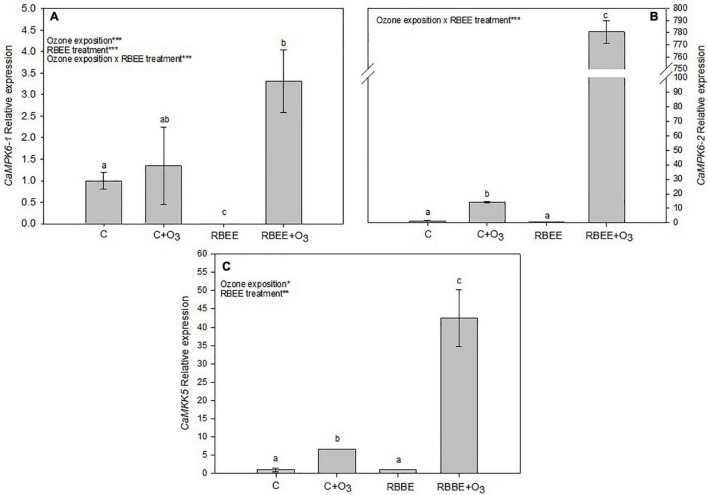
Relative expession of **(A)** *CaMPK6-1*, **(B)** *CaMPK6-2*, and **(C)** *CaMKK5* in leaves of pepper plants in response to two conditions of ozone (O_3_) (0 and 100 ppm) under a treatment without and with rice bran enzymatic extract (RBEE). For each gene, the expression level is relative to that of control plants (without RBEE treatment and without O_3_ exposition) considered as 1. Values represent relative expression ± SD, *n* = 3. Different letters indicate means that are significantly different from each other (two-way ANOVA, O_3_ exposition × RBEE treatment; HSD test, *P* < 0.05). O_3_ exposition, RBEE treatment, and O_3_ exposition × RBEE treatment in the corner of the panels indicate main or interaction significant effects (*P < 0.05; **P < 0.001; ***P < 0.0001).

This study also checked the expression of some MAPK genes involved in oxidative stress in plants (Liu and He, 2017). As suspected, expression of *CaMPK6-1*, *CaMPK6-2*, and *CaMKK5* genes increased in leaves of O_3_-exposed plants (Figure 5). RBEE did not give rise to the expression of these genes in plants in the absence of an O_3_ environment. Surprisingly, however, after O_3_ exposure, RBEE treated leaves showed a huge overexpression of these MAPK genes, these being expressions of 4.89, 55.31, and 6.39-folds higher than in the O_3_-exposed control plant for *CaMPK6-1*, *CaMPK6-2*, and *CaMKK5* genes, respectively (Figure 5). This result could indicate that the O_3_-oxidated RBEE produces the induction of gene expression by acting as a transcription inductor. However, this fact should be studied to understand the mechanism involved in this process.

## Discussion

Biostimulants have been defined as “a formulated product of biological origin that improves plant productivity as a consequence of the novel, or emergent properties of the complex of constituents, and not as a sole consequence of the presence of known essential plant nutrients, plant growth regulators, or plant protective compounds” (Yakhin et al., 2017). Present results show a new product, an enzymatic extract from rice bran that protects against O_3_-induced damage. O_3_ treatment induces a decrease in net photosynthetic rate, chlorophyll content, and DF in pepper leaves which was partially reversed after foliar treatment with RBEE (Figures 1A,B, 2A, respectively). Thus, we can propose that RBEE may exert a biostimulant effect on pepper plants mainly based on its bioactive compounds.

Rice bran enzymatic extract, the enzymatically obtained extract from rice bran, shows a notable total antioxidant capacity (Revilla et al., 2009) and has been evaluated as a protector against lipid and protein oxidation in rat brain homogenate (Parrado et al., 2003). Additionally, we tested the antioxidant capacity of RBEE in two cell models: keratinocyte monolayers and reconstructed human epidermis both irradiated with UVB, and found that RBEE decreased lipid peroxidation in both systems (Santa-María et al., 2010).

Rice bran enzymatic extract is rich in prominent bioactive molecules. Many components of the extract may contribute to its antioxidant capacity. The most abundant phytochemical is γ-oryzanol, which is a natural antioxidant composed of ferulic acid esters of sterols and triterpene alcohols with high scavenging activity of free radicals, mainly mediated by ferulic acid moiety (Lemus et al., 2014; Minatel et al., 2016; Massarolo et al., 2017; Zduńska et al., 2018). RBEE also contains tocols (both tocopherols and tocotrienols) that act as antioxidants due to their ability to donate phenolic hydrogens (electrons) to lipid radicals (Xu et al., 2001). Polyunsaturated fatty acids which act as radical scavenging agents are also present in RBEE, contributing to the antioxidant activity. So, other natural substances such as *Rosa rubiginosa* oil (Franco et al., 2007), grape seed oil (Bail et al., 2008), and soybean-germ oil (Chen et al., 2019) are used for cosmetic purposes due to their polyunsaturated fatty acid content, which acts as a radical scavenging agent.

Rice bran enzymatic extract is also rich in PHs. PHs exhibit different antioxidant and free radicals scavenging activities (Li et al., 2008), mainly conferred by some nitrogenous compounds contained therein such as glycine, betaine, and proline (du Jardin, 2015). Moreover, PHs have shown an ability to enhance antioxidant mechanisms in plants (Gurav and Jadhav, 2013). It is worth noting that PHs, mainly those resulting from the enzymatic hydrolysis of protein substrates into low molecular weight peptides and free amino acids, have shown multiple biostimulant capabilities. Protein hydrolysates are included within the biostimulant classification (du Jardin, 2015). The direct effects in plants include modulating N uptake and assimilation, acting on signaling pathways in the root, regulating enzymes involved in this process (du Jardin, 2015), possessing hormonal activity similar to auxin and gibberellin (Colla et al., 2014), and producing antioxidant activity (Li et al., 2008; Gurav and Jadhav, 2013). In addition, when applied to soils, PHs have shown indirect effects on plant growth and nutrition by increasing the availability of nutrients and their acquisition by the roots – enhancing the microbial activity and biomass of the soil, soil respiration, and their fertility in general (du Jardin, 2015; Rodríguez-Morgado et al., 2015).

It is worthing to note that mixture of these bioactive molecules, as is the case of RBEE, has been reported to greatly enhance the antioxidant activity of rice bran oil (Perez-Ternero et al., 2017).

According to the RBEE antioxidant capacity, we have selected an acute O_3_ treatment as an abiotic stressor. O_3_ is degraded in the apoplast into secondary ROS (Vainonen and Kangasjärvi, 2015) and high levels of ROS can lead to ROS stress that induces the enzymatic and non-enzymatic-systems that protect cells from ROS (Sachdev et al., 2021). Accordingly all the enzymatic activities assayed – CAT, GPX, APX, and SOD – were induced after O_3_ treatment. Induction was significantly reversed by the foliar treatment with RBEE (Figure 3). The effect on enzymatic activities could be correlated with the antioxidant effect of RBEE, thus the lipid peroxidation induced by O_3_ treatment was significantly alleviated by RBEE (Figure 4A). Consequently, the antioxidant effect is reflected in the OSI values (Figure 4B).

Unlike other enzymes assayed, a surprising result was the induction of APX after the application of RBEE in plants not treated with O_3_ (Figure 3B). We can speculate that this APX induction may be due to a hormetic effect induced by RBEE, stimulating a cellular system that could be essential for plant defense under stress. At the physiological level, hormesis is an adaptive response of an organism to a low-level stress factor accompanied by over-compensation when homeostasis is interrupted (Mattson, 2008; Calabrese, 2009; Wiegant et al., 2012). Hormesis is the cellular response in plants that occurs after an initial exposure to low levels of biotic or abiotic stressors – such as herbicides, temperature, chemicals, and radiation – which predisposes them to stimulate cellular defense mechanisms at subsequent sources of stress (Agathokleous and Calabrese, 2020; Berry and López-Martínez, 2020). For example, low doses of some herbicides such as 2,4-dichlorophenoxyacetic acid, glyphosate, and paraquat have been shown to trigger auxin production and antioxidant defense. The biostimulant-induced hormesis response of beneficial organisms allows plants to tolerate stress (Ahkami et al., 2017) through activation of secondary metabolisms and gene expression to recover homeostasis (Vargas-Hernandez et al., 2017). Application of biostimulants at right time can therefore facilitate increased plant growth while combined application of multiple biostimulants can be effective in reducing environmental drastic impacts (Dong et al., 2020).

In plants, the mechanism of hormesis is still unknown. The most probable pathways for hormetic responses are the induction of ROS by mild stress which leads to the activation of antioxidant defenses, stress-signaling hormones, or adaptive growth responses (Poschenrieder et al., 2013). Thus, induction of low and sub-toxic concentrations of ROS by mild stressors, such as which occurs as a result of foliar application of RBEE, has shown the ability to develop a hormetic effect, activating antioxidative defense and adaptive responses. RBEE is rich in 18C-unsaturated fatty acids (UFAs): oleic (18:1), linoleic (18:2), and α-linolenic (18:3) acids (Table 2). Besides their roles as ingredients and modulators of cellular membranes, reserves of carbon and energy, stocks of extracellular barrier constituents (e.g., cutin and suberin), or precursors of various bioactive molecules (e.g., jasmonates and nitroalkenes), recent works have pointed the role of 18C-UFAs as regulators of stress signaling (He and Ding, 2020). Oleic acid has been implicated in plant immunity against pathogens (Mandal et al., 2012). Even more, linoleic acid may regulate plant defense through ROS production (Yaeno et al., 2004). In this context, we can assume that RBEE application may induce a mild increase in ROS due to UFAs content that underly the induction of APX activity.

Ascorbate peroxidase is an H_2_O_2_-scavenging enzyme and is indispensable for the protection of chloroplasts and other cell constituents from damage by H_2_O_2_ and hydroxyl radicals (OH.). APX has been identified in most higher plants and comprises a family of isoenzymes present in different plant cell compartments, including apoplast (Sofo et al., 2015). As compared to catalase, it is more vital in stress as it has a high affinity for hydrogen peroxide (Afzal et al., 2014). Accordingly, we have found that APX activity exceeds catalase activity 10 times (Figures 3A,B). After O_3_ enters the leaves through the stomata, it rises to the apoplast where it is immediately degraded into secondary ROS. Detoxification of ROS in the apoplast can therefore be considered as an early line of defense against O_3_ (Kangasjärvi et al., 2005; Castagna and Ranieri, 2009). APX uses ascorbate as its specific electron donor to reduce H_2_O_2_ to water. Ascorbate is believed to be the major redox buffer and ROS scavenger in the apoplast (Foyer and Noctor, 2009). Thus, we can speculate that pretreatment with RBEE induced ROS generation that slightly stimulates APX activity, probably at the apoplast, the gate of entrance, that facilitates posterior induction and consequently protection against O_3_ exposure.

The APX result could be working in tandem with the decrease in chlorophyll content and DF found in the same group of plants (Figures 1B, 2A). This effect could be due to linoleic acid present in RBEE as it has been shown that linoleic acid decreased the chlorophyll concentration and photosynthetic efficiency in *Cylindrospermopsis raciborskii* (Xu et al., 2016).

Protein phosphorylation mediated by protein kinase cascades is one of the most important post-translational modifications that coordinate response in cells. One relevant protein-kinase based amplification cascade is the MAPK cascade. The MAPK cascade is a complex signaling pathway hierarchically organized at least three sequentially acting serine/threonine kinases – a MAP kinase kinase kinase (MAPKKK), a MAP kinase kinase (MAPKK), and finally, the MAPK itself – with each phosphorylating, and hence activating, the next kinase. In this cascade, MAPKs phosphorylate specific substrate proteins, such as transcription factors and enzymes, and subsequently trigger cellular responses and rapidly transform upstream signals into appropriate intracellular responses (Liu et al., 2015). MAPK cascade is involved in many aspects of plant physiology, including cell division (Komis et al., 2011), plant growth and development (Xu and Zhang, 2015), plant resistance to pathogens (Bigeard et al., 2015) and insect herbivores (Hettenhausen et al., 2015), and plant response to abiotic stresses (Liu, 2012; Smékalová et al., 2014). It has been described as an interplay between MAPK cascade and ROS; so exogenous application of H_2_O_2_ or O_3_ activates components of MAPK cascades. On the other hand, manipulating MAPK cascades results in initiation of ROS responses (Šamajová et al., 2013). In this context, the present work analyses the role of the MAPK cascade in the protective effect of RBEE against O_3_ damage.

The majority of MAPK and MAPKK members are constitutively expressed in pepper plants. In leaves, the transcript level of *CaMPK1* is the highest, followed by *CaMPK6-2*, and *CaMPK19-2*. Among MAPKK genes, *CaMKK5* exhibits the highest transcript levels. Under the challenges of heat shock, salt stress, or pathogen inoculation, most of the MAPKs and MAPKKs in the pepper genome were significantly transcriptionally modified (Liu et al., 2015). Accordingly, after O_3_ exposure, we found induction of the studied MAPKs. It is worth noting the relevant role that MPK3/MPK6 play in ROS response and O_3_ sensitivity. AtMPK3/AtMPK6 in *A. thaliana* (Ahlfors et al., 2004) and the orthologs SIPK/WIPK in tobacco (Marcus et al., 2000), have been found to regulate for O_3_ sensitivity; in fact RNAi-mediated silencing of MPK6 renders the plant more sensitive to O_3_ (Miles et al., 2005). In these lines we found increased CaMAPK6-1 and CaMAPK6-2 after pepper plants exposition to O_3_. Moreover, induction of CaMKK5 may be related to CaMAPK6-1 and CaMAPK6-2. It has been described in *Arabidopsis* that MKK5 was involved in MPK3/MPK6 activation in response to O_3_ exposure (Vainonen and Kangasjärvi, 2015) and interestingly in pepper CaMPKK5 interacts with CaMPK3, CaMPK6-1, and CaMPK6-2 (Liu et al., 2015).

But surprisingly, after O_3_ exposure, RBEE treated leaves showed a huge expression of these MAPK genes, being higher than in the control plants under O_3_ in *CaMPK6-1*, *CaMPK6-2*, and *CaMKK5* genes (Figure 5). As previously indicated, the MAPK cascade is involved in many aspects of plant physiology including the defense response against stress. The presence of multiple genes’ family members in genomes of different plant species encoding for MAPKKKs, MAPKKs, and MAPKs, and the fact that one MAPK cascade may be associated with more than one upstream or downstream partner (Liu et al., 2015) made it difficult to elucidate specific mechanism mediated by MAPK modules. How ROS activates MAPK cascades still remains unclear. It is possible that plants not only use MAPK cascades to transduce ROS signaling to gene expression and sometimes cell death, but also initiate the negative feedback regulation by MAPK cascades to maintain ROS homeostasis. The different combinations of the three tiers of kinases, distribution, time point-dependent activation, strength, duration, and availability of substrates of MAPK cascades may determine the feed-forward or feed-back outcomes (Liu and He, 2017). However, our results unequivocally indicate that both the damage induced by O_3_ and the activation of the protection systems against it induced by RBEE take place through the transcriptional induction of the MAPK cascade.

## Conclusion

Rice bran enzymatic extract, an enzymatic extract of plant origin, reversed the O3-induced decrease in physiological parameters as net photosynthetic rate, chlorophyll content, and DF. Thus, the results of our study highlight the potential use of RBEE as an effective biostimulant plant protector against oxidative stress caused by O_3_. Present results also point out that MAPK cascade is involved in both, O_3_-induced damage and RBEE protection. However, more studies are needed to clarify how this kinase pathway is involved.

Thus, we contribute to the general efforts done in the last decade searching new non-chemical alternative products to protect crops against damages caused by environmental oxidative stress.

## Data Availability Statement

The original contributions presented in the study are included in the article/Supplementary Material, further inquiries can be directed to the corresponding author.

## Author Contributions

SM-B, SN-T, ER, AC, and JP designed the study. SM-B, SN-T, and LM performed the research. SM-B, SN-T, PC, ER, AC, and JP analyzed the data and wrote the manuscript. All authors contributed to the article and approved the submitted version.

## Conflict of Interest

The authors declare that the research was conducted in the absence of any commercial or financial relationships that could be construed as a potential conflict of interest.

## Publisher’s Note

All claims expressed in this article are solely those of the authors and do not necessarily represent those of their affiliated organizations, or those of the publisher, the editors and the reviewers. Any product that may be evaluated in this article, or claim that may be made by its manufacturer, is not guaranteed or endorsed by the publisher.
