# Supplementary material for: Biostimulant Capacity of an Enzymatic Extract From Rice Bran Against Ozone-Induced Damage in Capsicum annum
Source: Front Plant Sci. 2021 Nov 19;12:749422. doi: 10.3389/fpls.2021.749422 (PMC8641545; doi:10.3389/fpls.2021.749422)
Supplement: Supplementary file 1 [file Data_Sheet_1.docx]

| MW | % |
| --- | --- |
| > 10 KDa | 7.27 ± 0.6 |
| 10-5 KDa | 2.68 ± 0.1 |
| 5-3 KDa | 2.85 ± 0.1 |
| 3-1 KDa | 11.56 ± 2.0 |
| 1-0,3 KDa | 26.50 ± 2.6 |
| > 0,3 Kda | 49.13 ± 1.4 |


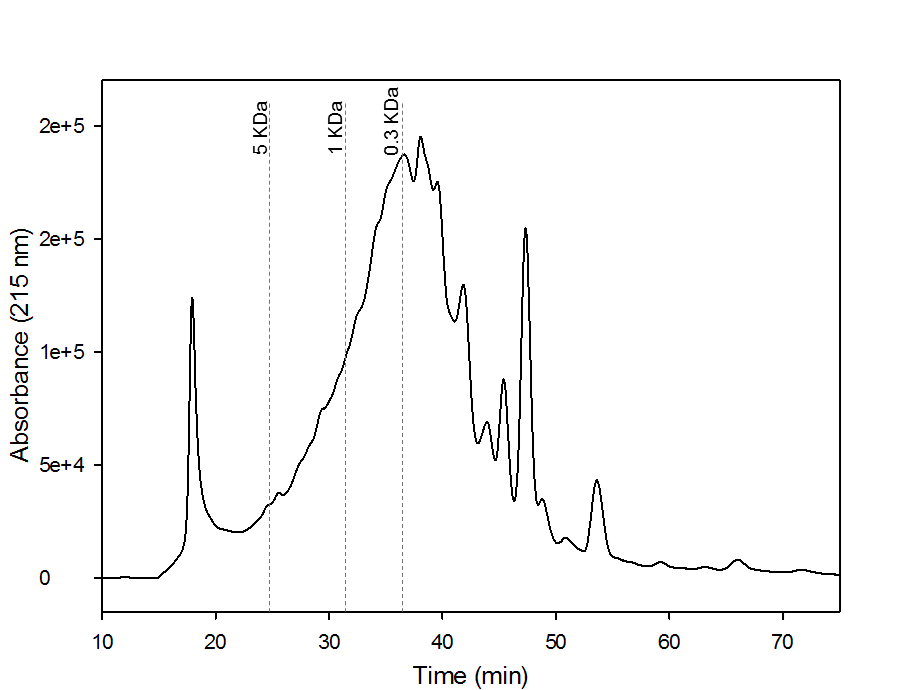


Figure SM1. Molecular weight profile of the soluble organic component of RBEE (Chromatogram obtained by molecular exclusion HPLC, 215 nm). The results are mean values of three analyzes.


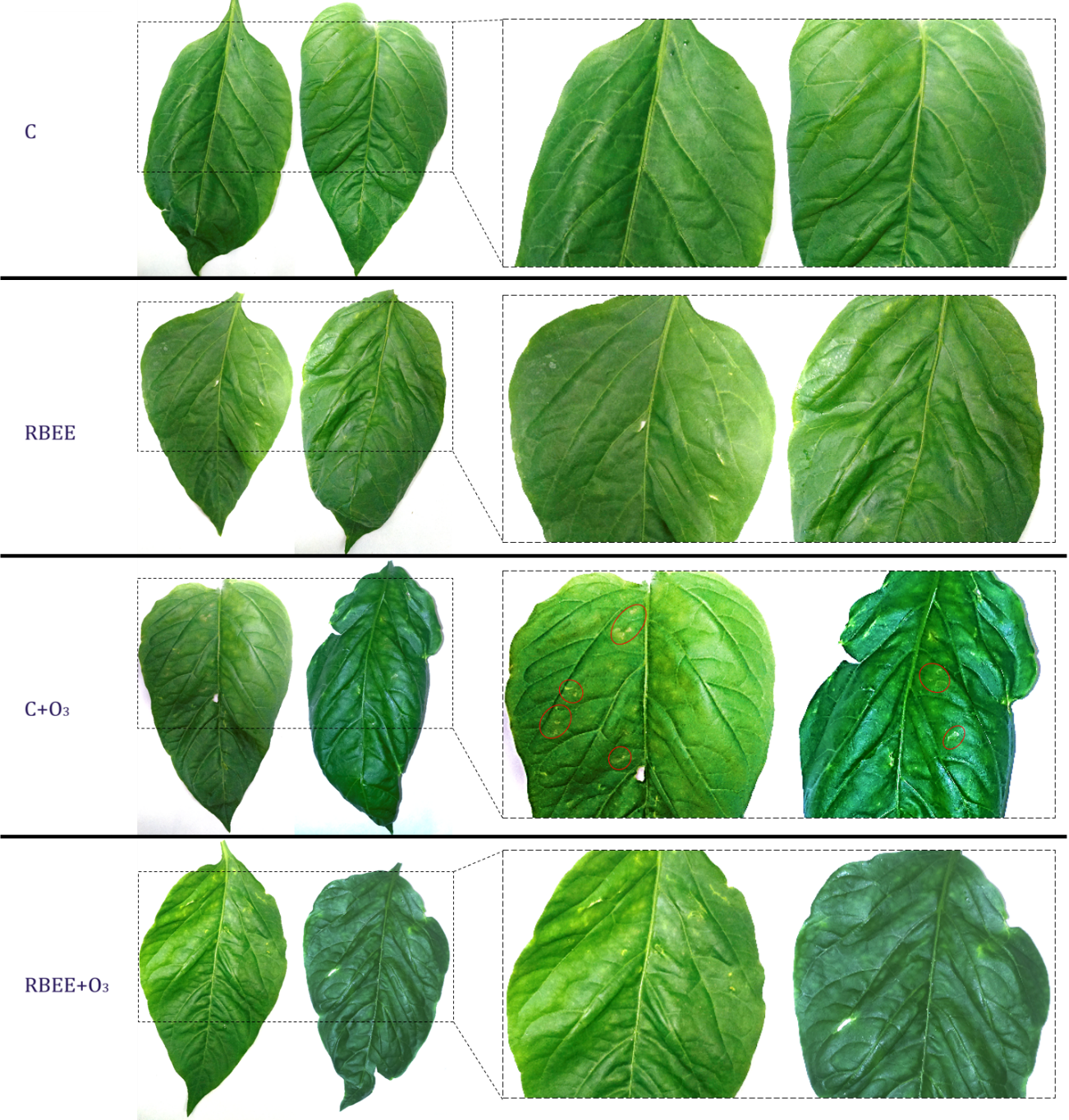


Figure SM2. Images showing chlorosis in ozone-exposed plants. C+O_3_ plants showed a more widespread chlorosis, as well as the appearance of small brown spots that were not present on RBEE+O_3_ plants. The tiny brown spots have been indicated by circling them in red.
